# “It’s a Postcode Lottery”: How Do People Affected by Dementia in Wales Experience Their Diagnosis and Post-Diagnostic Support, and How May These Be Improved?

**DOI:** 10.3390/ijerph21060709

**Published:** 2024-05-30

**Authors:** Jennifer Rhiannon Roberts, Gill Windle, Catherine Anne MacLeod, Mary Pat Sullivan, Paul M. Camic, Joshua Stott, Emilie Brotherhood, Kiara Jackson, Sebastian Crutch

**Affiliations:** 1 DSDC Wales Research Centre, School of Health Sciences, Bangor University, Ardudwy, Normal Site, Bangor LL57 2PZ, UK; g.windle@bangor.ac.uk (G.W.);; 2 Faculty of Education and Professional Studies, School of Social Work, Nipissing University, North Bay, ON P1B 8L7, Canada; 3 Dementia Research Centre, Queen Square Institute of Neurology, University College London (UCL), London WC1N 3AR, UK; 4 Department of Clinical, Educational and Health Psychology, University College London (UCL), London WC1E 6BT, UK

**Keywords:** dementia, public health, dementia strategy, rarer dementias, rural, lived experience, diagnosis, post-diagnostic support

## Abstract

National dementia strategies are government policies that guide the provision of appropriate support for people living with dementia. These strategies, developed through extensive stakeholder engagement, should be tailored to the cultural and demographic needs of a country. Using a mixed methods survey design, this study explored the aims of the Dementia Action Plan (2018–2022) for Wales (UK) around assessment, diagnosis, and post-diagnostic support, and assessed whether these are being realized. Further, it sought to gain insight from people living with dementia and their carers around how the experience may be improved for others in the future, as the development of the next iteration of the Action Plan is anticipated. Respondents included 71 people, affected by typical and rarer types of dementia, living in both rural and urban areas. Findings suggest both positive and negative experiences, reflecting a ‘postcode lottery’ of service provision. Attainable recommendations for improvement were made by respondents, which would ultimately likely be cost-effective and reduce strain on formal services. The findings reported in this paper concur with those reported by people living with dementia in other countries, indicating their relevance for policymakers beyond Wales.

## 1. Introduction

Disclosing and receiving a diagnosis of dementia is generally a negative process for all involved (people with dementia, carers, and health care practitioners [HCPs]; [1]). For HCPs, the process is difficult, complex, emotionally burdensome, and underpinned by a lack of formal training on how best to deliver the diagnosis. For the person receiving the diagnosis as well as their families, it is associated with negative emotions such as shock, depression, anxiety, fear, uncertainty, and a loss of hope [1]. However, receiving a higher-quality diagnosis disclosure has been associated with lower levels of depression and despair, as well as higher levels of acceptance and reassurance following diagnosis [2]. This is an encouraging finding with important implications for policy and practice.

In England and Wales [3], as well as internationally [4], guidelines around assessment, management, and support of people living with dementia and their carers advocate for referrals to memory clinics or specialist clinicians for a comprehensive assessment and diagnosis of dementia; however, these services may not always be readily accessible. Services for people living with dementia may differ across localities, essentially leaving people’s fate to a ‘postcode lottery’ for service quality and quantity [5]. This issue may be exacerbated in rural areas, rendering diagnosis and post-diagnosis support dependent on the skills, knowledge, and expertise of the primary care providers/general practitioners (GPs) local to a particular area [6]. This challenge of service disparity is an international one, with similar challenges reported in England [7], Denmark [8], the United States of America [9], Canada [10], and Australia [11].

The recent scoping review by Roberts et al. [6] found that difficulties in obtaining a diagnosis and limited access to specialists in rural areas is an international public health concern. Innes, Szymczynska, and Stark [12] explored the diagnostic process for people living with dementia and carers in rural Scotland, finding variability in the time taken to receive a diagnosis and a desire by participants for more information about the process to help them prepare. How the diagnosis is disclosed was identified as important, also having an effect thereafter on post-diagnostic support experiences. A report from the Alzheimer’s Society in Wales [13] also highlights the issues faced by people affected by dementia in rural areas. Participants revealed GPs as being their first point of contact for concerns about symptoms, and while often accessible, they were often slow in diagnosing dementia and referring people to memory assessment/specialist services. Moreover, some suggested that their GP was not able to identify differences between symptoms of ‘normal aging’ and dementia; an issue that was more apparent in people living with young-onset dementia. This suggests that problems around diagnosis may be exacerbated further still for people whose symptoms do not conform to those associated with more ‘typical’ forms of dementia and supports findings around difficulties during the diagnostic process internationally for people living with young-onset dementia [14]. In England and Wales, prior to referral to specialist dementia diagnostic services, such as memory clinics for a formal diagnosis, most people concerned about their symptoms will visit a GP. Guidance around initial assessment in non-specialist settings such as General Practice [3] includes taking a history (including symptoms, and their impact on daily life) both from the person and somebody close to them, and not ruling out dementia only because a person has a normal score on a cognitive instrument. However, this again relies on the person carrying out the assessment being equipped with the education and resources to identify possible signs of younger onset and rarer forms of dementia. Rare dementias are characterized as atypical and inherited forms of dementia often being younger at onset and including symptoms other than memory loss, such as those defined by Rare Dementia Support [15].

Beyond the diagnosis, difficulties and inequities in accessing post-diagnostic support are common, particularly for those living in rural areas [6,16,17]. Further, for people living with rarer forms of dementia, access to post-diagnostic support is often delayed, and where services are available, they are often deemed unsuitable [10,14,18,19].

Wales has a large proportion of rural areas, with around one-third of the country classed as rural [20,21]. It is estimated that 46,800 people are living with a diagnosis of dementia in Wales [22], and an estimated 17,000 of those live in rural areas [23]; this high proportion is presumed to be due to a greater rural population than other parts of mainland Great Britain, coupled with the rapidly growing aging populations in rural areas [23]. This reveals the importance of including the experiences and perceptions of people affected by different types of dementia in rural areas in developing and improving support going forward.

National dementia strategies are government policies developed to guide the provision of appropriate support for people living with dementia. Through extensive stakeholder engagement, they should be tailored to the specific cultural and demographic needs of a country. Key actions that are common between countries include raising awareness of dementia, reducing stigma, assessing, and improving access to diagnostic services, identifying post-diagnostic support services, and improving the quality of care [24].

The Welsh Government has devolved responsibility for health and social care provision. A Dementia Action Plan for Wales was developed in 2018 by the Welsh Government [25]. Regarding the diagnosis experience specifically, the plan states:

*“We have the right to an early and accurate diagnosis, and to receive evidence based, appropriate, compassionate and properly funded care and treatment, from trained people who understand us and how dementia affects us. This must meet our needs, wherever we live.”*

[25] (p.10)

The Action Plan sets out aims for improving assessment and diagnosis, as well as plans for post-diagnostic support services that support people to live as well as possible, for as long as possible. However, according to a recent survey by the Alzheimer’s Society in Wales [26], only 19% of people affected by dementia were offered an assessment and support plan at the time of diagnosis. These findings suggest that there may be a way to go before the “vision for Wales to be a dementia friendly nation” [25] (p.3) is realized.

As the timeline (2018–2022) for the Dementia Action Plan came to an end, a bridging plan was published [27] whilst the priorities for its successor’s arrangements are identified. This bridging policy focuses on strengthening priorities from the existing Action Plan that have been particularly impacted by the COVID-19 pandemic, for example, assessment and diagnosis, timely diagnosis (within 12 weeks), the learning and development needs of both health and care practitioners, and unpaid carers, and living as well as possible for as long as possible. The All-Wales Dementia Care Pathway of Standards [28] further supports the implementation of the Action Plan, aligning with its aims. They promote a whole systems integrated care approach for advancing dementia care in Wales and provide a set of standards that each region in Wales is funded to work towards.

### Aims

This study explored the aims of the Dementia Action Plan around the two themes of assessment and diagnosis, and post-diagnostic support across Wales, and assessed whether these are being realized. Further, it sought to gain insight and recommendations from people living with dementia and their carers, specifically around how the experiences may be improved across Wales, as we anticipate the development of the next iteration of the Action Plan.

The research questions are as follows:

(1) How do people affected by dementia in Wales experience the diagnosis and post-diagnostic process in relation to the aims outlined in the Action Plan?
(2) Does this differ as a function of their location of residence (rural vs urban) or their diagnosis type (typical vs. rare forms of dementia)?
(3) How can the diagnosis and post-diagnostic process in Wales be improved?

## 2. Methods

### 2.1. Participant Recruitment

An online survey was open to anyone over the age of 18 living with or caring for someone with any type of dementia, diagnosed in any part of Wales, at any point in time. The study was promoted in several ways. A poster together with further information advertising the study was shared through The Centre for Ageing and Dementia Research (CADR Cymru), and North and Mid-Wales Dementia Networks via email and social media. These communities have a combined membership of over 1000 people who have an interest in dementia, either personally or professionally. Further, the study was shared with members of DEEP, the UK network of dementia voices (www.dementiavoices.org.uk, accessed 15 August 2023), and members of the Rare Dementia Support network living in Wales (www.raredementiasupport.org, accessed 15 August 2023). The survey was shared on the social media platforms Facebook and Twitter. A press release also promoted the work. The survey was available online from May to November 2022. We aimed to recruit as many people as possible but with a target of 100.

### 2.2. Ethics

This is a sub-study of the larger Rare Dementia Support (RDS) Impact Project [29], with ethical approval granted by the University College London Research Ethics Committee (8545/004: RDS Impact Study). Brief study information was given on the first page of the survey, as well as a link to the full participant information sheet as outlined in the approved study protocol. Participants were asked to proactively opt-in to proceed if they could confirm they were eligible to participate and that they consented to take part.

### 2.3. Survey Design

Development of survey items took place over two months. Items were drafted by the lead author and shared with all co-authors for feedback. Furthermore, feedback was sought from other professionals with an interest in improving dementia support in Wales including a policy officer from a third-sector organization, a consultant nurse for dementia, and a local authority dementia project manager. Items were discussed and refined with input from all co-authors. Once finalized, the questionnaire was translated into Welsh by JR (a native Welsh speaker) and proofread by the University’s translation unit. The questionnaire is available in the Supplementary Materials.

This study adopted a mixed methods design, and data were collected via the online survey platform Qualtrics. The quantitative element explored experiences relating directly to the aims of the Dementia Action Plan, and a qualitative element allowed participants to elaborate on their experiences, providing the research team the opportunity to identify themes additional to those presented within the Action Plan. The survey was available in English or Welsh and could be completed by people living with dementia or carers/family members, with an initial question ascertaining which of these a respondent was. The wording was suited to the respondent (e.g., ‘Do you have a personal care plan?’ [people with dementia], and ‘Does the person with dementia have a personal care plan?’ [carers/family members]).

Demographic data were obtained including age, gender, marital status, ethnicity, occupation, living arrangements of a person with dementia, rural or urban residency of a person with dementia, relationship to the person with dementia (carers), and whether a carer’s need assessments were made (carers). Another set of items sought details around the diagnosis such as dementia type, time since diagnosis, when symptoms were first noticed, reason for seeking help, the referral pathway, and length of time to obtain the diagnosis. The rest of the questionnaire was developed to align with the aims of the Dementia Action Plan around diagnosis and post-diagnostic support (themes 4 and 5 within the Action Plan) with questions including binary yes/no questions, multiple choice questions, and Likert scales. A total of 43 items relating to the aims of the Action Plan were included.

A further 7 open-ended questions explored people’s spontaneous responses around what they had found most helpful, and most difficult, what could be done differently in the future, what they believe would have been the most useful support to be offered after diagnosis, anything that they would like to see improved, anything about the delivery of the diagnosis that they would change, and anything else that they felt was important to share. Welsh responses were translated by JR for analysis and reporting.

The rationale for using the survey method was that it may attract a wide range of respondents, particularly those who may not be familiar with participation in research studies. We anticipated that an online survey might offer the potential opportunity to gain a broader understanding from a larger sample than restricting the study to qualitative methods and may be less burdensome than undertaking in-depth interviews.

### 2.4. Quantitative Analysis

Survey data were analyzed using SPSS version 29 (IBM Corp., Armonk, NY, USA). Descriptive statistics explored the frequencies of responses. To explore differences as a function of the location of residence and dementia type the following criteria were employed: Typical dementias were defined as Alzheimer’s disease, vascular dementia, mixed Alzheimer’s, and vascular dementias, or those where no specific diagnosis was given but other responses indicated a memory-led dementia in people over 65; all other diagnoses, including familial variants, young onset and non-memory-led dementias were defined as rare dementias [15]. In terms of rurality, a question around the location of residence gave the options of rural, urban, or other; ‘other’ allowed the respondent to specify and in these instances (*n* = 10), ‘semi-rural’, ‘village’ and ‘near to a small town’ were combined within the ’rural’ category, and ‘town’ and ‘suburban’ were combined within the ‘urban’ category.

For dichotomous items, Chi-squared tests were used to examine whether there were differences in responses as a function of the location of residence (rural vs urban) and dementia type (rare vs typical), and p-values were calculated. Where expected frequency counts were less than five, Fisher’s exact test was used. Likert scale responses (5-point) were collapsed into three categories by combining ‘strongly agree and agree’ and ‘strongly disagree and disagree’ into single variables, alongside the neutral ‘neither agree nor disagree’ response, before performing Chi-square analyses to determine any differences between independent variables.

### 2.5. Qualitative Analysis

Qualitative analysis was conducted using NVivo (version 12) (Lumivero, Denver, CO, USA) via secure remote access. Thematic analysis [30] of all open-ended question entries was carried out by two researchers (JR and KJ). JR and KJ generated initial codes independently of one another through reading and reflecting on all responses by all respondents adopting an inductive approach. They then compared and contrasted codes. Codes were discussed and adapted, and final themes were agreed upon through discussions between JR and KJ. Themes were then categorized into difficulties/negative experiences, positives/helpful experiences, and recommendations/requirements.

## 3. Results

**Figure 1:**
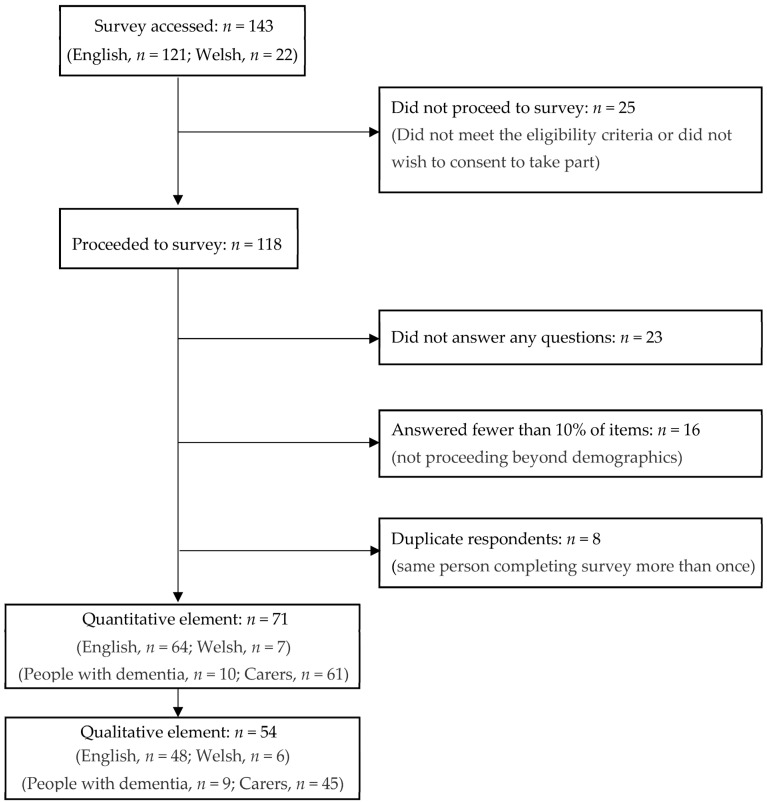
Flow through the survey by respondents.

The study flow is indicated in Figure 1. Of the 143 people accessing the survey, 71 (64 English, 7 Welsh) completed at least 40% of the study, comprising people living with dementia (*n* = 10) and carers/family members/bereaved carers (*n* = 61).

Table 1 provides a breakdown of demographic and diagnosis information of the people living with dementia whose experiences the survey explores (both from people living with dementia who completed the questionnaire themselves (*n* = 10), and carers or family members (*n* = 61), and demographic information about the carers completing the study. The specific diagnoses of people living with dementia included 17 people with rare forms of dementia and 54 with more typical forms. In terms of location of residence, 8 people with rare forms of dementia lived in rural areas, and 9 in urban; and 29 people with typical forms of dementia lived in rural areas, and 28 in urban areas.

Table 2 provides an overview of the diagnosis experience of respondents. Most were diagnosed within the timeframe of the Action Plan, and the diagnosis process from first visiting the GP to receiving the diagnosis for the majority was more than 3 months (12 weeks). Worries about memory and family concerns were the main reasons for seeking help, and few reported having a full medical history taken during that first GP visit (as per NICE guidelines [3]). Other reasons for contacting the GP initially included hallucinations, depression, difficulties with language production (word loss, stammer), and a feeling that something ‘wasn’t right’. For some, the journey to diagnosis began through contact with different health professionals, including treatment for depression by a mental health team, a nurse who noticed a difference in the person during a blood test, post- hospital admissions, and peripheral vision difficulties identified by an optician.

Referrals for most from the GP were to the memory clinic, as would be expected given the higher proportion of people affected by more typical dementias who took part. Others were referred to a neurologist, Community Psychiatric Nurse, Parkinson’s specialist, and the local hospital eye clinic. One person said they were ignored by their GP despite clear evidence of dementia, and another said that their symptoms were dismissed as depression until they insisted on a referral to a memory clinic. In terms of the diagnosis itself, the experience of the majority was that it was delivered face-to-face, a family member accompanied them to the appointment, and it was provided both verbally and in writing. Despite this, nearly a third reported not receiving the diagnosis both verbally and in writing, with some attending their appointment alone.

**Figure 2:**
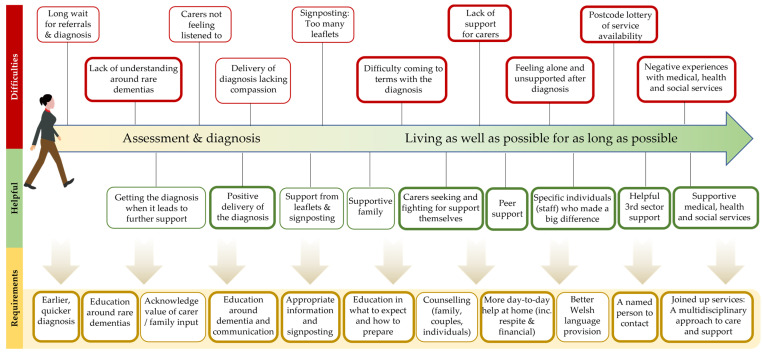
A visual representation of the themes that emerged from the qualitative element of the study.

Figure 2 provides a visualization of the themes emerging from the thematic analysis of the qualitative element of the study completed by fifty–four people. Bold boxes indicate themes that were mentioned 10 or more times (range 10–32). These themes describe both helpful and difficult experiences of diagnosis and post-diagnostic support. The qualitative themes are described below together with findings from the quantitative data analysis. In the quantitative element of the study (*n* = 71 respondents), very few significant differences were found as a function of location of residence or dementia type. Where significant differences were observed, these instances are indicated in the tables and described within the text.

### 3.1. Assessment and Diagnosis (Dementia Action Plan Section 4)

Table 3 provides personal perspectives around the assessment and diagnosis, in terms of agreement or disagreement with statements relating to the aims of the Action Plan. No significant differences were found as a function of location of residence or dementia type (available in Supplementary Material).

#### 3.1.1. Being Diagnosed vs. Long Wait for Referrals and an Accurate Diagnosis

Almost half (45%) of respondents agreed that the diagnosis was given at the right time. The qualitative analysis included reports of people having to wait weeks, months, and years for referrals and diagnoses. Some carers reported not feeling listened to and said that their GP and other professionals did not listen, understand, or believe them when they raised concerns about their loved one’s health, which contributed to the difficulty in receiving a diagnosis and support thereafter. Others said that receiving the diagnosis was seen as helpful and had led to further support.

*“Not being believed by GP”*.

(Carer of a person with a typical form of dementia)

In terms of accessibility, most respondents were able to access the GP, hospital, and memory service close to home (either within 10 miles or 10-20 miles away). However, people affected by rare dementias had to travel significantly further to access specialist centers (z = −2.48, *p* = 0.013). Additional difficulties were also reported by people affected by rare dementias. Health professionals' lack of understanding and awareness around rare dementias led to misdiagnoses, long and complex journeys to diagnosis, and no specific help being provided thereafter.

*“GPs understanding more—3 years from referral to diagnosis was hard”*.

(Carer of a person with a rare form of dementia)

*“Earlier diagnosis. Took 10 years to confirm diagnosis, wrongly diagnosed with depression”*.

(Person living with a rare form of dementia)

#### 3.1.2. Positive Delivery of the Diagnosis vs. Delivery of Diagnosis Lacking Compassion

A majority of respondents (61%) agreed that the diagnosis was given with empathy, and the qualitative data described teams and individuals involved in the diagnosis as being “wonderful”, “supportive”, “brilliant”, and “kind”. Moreover, 55% agreed that the person giving the diagnosis was helpful, 57% said everything was explained clearly, and 62% understood the symptoms. Several people also said that they would not change anything about the diagnosis delivery and some elaborated that it was given in a supportive and kind manner.

*“The process we went through was good and efficient. Everyone has been supportive”*.

(Carer of a person with a typical form of dementia)

However, the qualitative data also revealed a conflicting theme, with some reporting a lack of empathy and compassion in communicating the diagnosis. In some instances, the person with dementia was ignored (when a carer was present), and one diagnosis was delivered over Zoom (during the COVID-19 pandemic) in what the respondent had thought was going to be a routine consultation.

*“The hospital staff who delivered it were lacking compassion, did not address the person with dementia at all, and made us all angry and upset. That should all have been different”*.

(Carer of a person with a typical form of dementia)

#### 3.1.3. Signposting That Did Not Feel Appropriate

According to 34% of respondents, the person giving the diagnosis knew what support was available for them. After the diagnosis, 29% knew where to go for help, and 27% knew what was going to happen next. Regarding signposting, 82% had received leaflets and written information. However, the qualitative data indicated that respondents felt they had received an overwhelming amount of leaflets or books that felt inappropriate. One person reported benefiting from leaflets and signposting, but this was during an information session after the diagnosis where they had the opportunity to choose leaflets and exchange information with others.

*“After the diagnosis, we left the Mind [Memory] Clinic with two books, not knowing what to do next: No direct organization or phone number. Later that week we were contacted by the Dementia Coordinator, who again gave us lots of paperwork and left saying ‘you don’t need any help at the moment’. Quite honestly, I found the handouts/books/paperwork too overwhelming”*.

(Carer of a person with a typical form of dementia)

### 3.2. Living as Well as Possible for as Long as Possible (Dementia Action Plan Section 5)

Table 4 provides the personal opinions of respondents regarding the support received after a diagnosis of dementia. No significant differences were found as a function of location of residence or dementia type (available in Supplementary Material).

Table 5 provides a summary of the support received by respondents in relation to the aims of the Action Plan. Significant differences as a function of rurality and dementia type are marked within the table and elaborated on below (Supplementary Materials provide further details). 

#### 3.2.1. Feeling Supported vs. Feeling Alone and Unsupported

Carers described a general lack of support for them, which included difficulties receiving help to access support (e.g., lasting power of attorney, personal independence payment, and carer’s allowance). Difficulties in accessing suitable respite care were evident in both qualitative and quantitative data. Further, only 27% of carers had received an assessment of their own needs. Several carers described having to do their own research and ‘fighting’ to access suitable support.

*“I think that there needs to be some sort of available support system. As a carer I have had to find things out for myself. This is draining and makes life more of a struggle”*

(Carer of a person with a rare dementia)

*“I had to find out information myself and make initial contact to get help. Why isn’t this information given at point of diagnosis?”*.

(Carer of a person living with a typical form of dementia)

Responses indicated that 34% of people living with dementia had a dementia support worker (defined as one named person who can be contacted about the person's care or anything that they are worried about), and half of those (48%) said that they connect them to appropriate information and support. Qualitative analyses revealed that people often feel alone and unsupported after diagnosis, often being discharged once the diagnosis is made. Some respondents did not know what to do or who to contact, being left to ‘get on with it’, with very little ongoing support. Difficulties in coming to terms with the diagnosis and changing abilities were experienced, and some felt helpless. Despite this, only 9% of respondents had accessed a dementia helpline. Moreover, only 13% of respondents had received counseling support and the proportion was significantly higher (*p* = 0.02) for people living with rarer forms of dementia.

*“You are left to essentially get on with things with minimal/no support”*.

(Carer of a person with a typical form of dementia)

Conversely, there were also reports of supportive medical, health, and social services, and descriptions of helpful teams, groups, and individuals that have made a significant impact. Specific individuals within services and organizations who have provided excellent support and understanding were named by a number of respondents.

*“A new member of my memory clinic who sat and listened to me and understood, at last someone understood and took time out, [name of person] great young man, thank you”*.

(Person living with a typical form of dementia)

*“A fantastic CPN who has provided excellent support”*.

(Carer of a person with a typical form of dementia)

The qualitative data revealed praise for third-sector support (e.g., charities or voluntary groups), with descriptions of support, involvement, and advice from charities. Furthermore, 72% had received contact information for relevant charities.

*“NEWCIS [Northeast Wales Carers Information Service] has been the most helpful and understanding service”*.

(Carer of a person with a typical form of dementia)

*“RDS [Rare Dementia Support] have been amazing, but I didn’t find them early enough”*.

(Carer of a person living with a rare form of dementia)

The importance of support groups, peer support, and talking to others in a similar situation was emphasized in the qualitative element of the study, and 67% were given details of support groups. Furthermore, 54% felt a sense of belonging and being valued, and 62% agreed that they lived in a supportive environment where they felt valued and understood.

*“Joining [name of local charity group] was the best thing we did, now we have support from professionals and other families living with dementia. The friendships we have made have been life changing for Mum and I”*.

(Carer of a person living with a typical form of dementia)

#### 3.2.2. A ‘Postcode Lottery’ of Service Availability

An inconsistent ‘postcode lottery’ of service provision is highlighted, detailing a disparity in quality and/or quantity of support between health boards/counties. Some described negative experiences with medical, health, and social services. Difficulties included a lack of post-diagnosis support, limited contact and support from social services, traumatic experiences within hospital settings, lack of support or understanding from the GP, and a lack of services in the local area for people who live rurally. Around a quarter (27%) agreed that services were designed around them and their individual needs, and 51% had personal choice and control or influence over decisions about themselves.

*“My parents live on the boundary between 2 health authorities. The original one told my mother that it was ‘probably Alzheimer’s’ and gave us no proper diagnosis for 5/6 years. It was only when the boundaries were redrawn, and we went to [name of health board] that we got any kind of real/correct diagnosis”*.

(Carer of a person with a rare form of dementia)

Although few significant differences have been found in the statistical analyses between people who live in rural versus urban areas, when asked “Do you think your experience is difficult because of whether you live?” 25% of respondents (17/67) said ‘yes’, and 71% (*n* = 12) of those lived rurally. A significantly higher percentage of those living with rare experienced this difficulty. Difficulties reported by people in rural areas included there being no/few services and supports nearby, and criticisms of specific health boards. Two people had moved after the diagnosis to be closer to better support. Difficulties in urban places (*n* = 4) related to disability, being discharged after diagnosis, living between two countries, and one person who was not sure but suggested “resources”. Finally, there was an acknowledgment that the COVID-19 pandemic also prevented access to services for people in Wales.

*“After the diagnosis I moved us so that we were nearer to the facilities I knew we would need”*.

(Carer of a person living with a rare type of dementia)

#### 3.2.3. Support for Maintaining Independence and Quality of Life

Personal care plans were received by 26% of those diagnosed. Of those, 44% said the person with dementia felt involved in drawing up their care plan, whereas 88% of carers felt involved. The care plan reflected goals for 63% of those diagnosed, and 73% agreed that it was clear and easy to understand. However, only 33% agreed that their care plan was helpful.

Over half (56%) had support that helped them live their life, and 30% had received support to improve or maintain their quality of life (e.g., art, music, sport, reminiscence groups). Furthermore, 25% received support to help improve and maintain their memory, and 24% were given the opportunity to take part in research. 

In terms of practical support for living at home, 46% were advised about adjustments to their environment (e.g., home), and 20% had received support to help them live at home (e.g., home care, meals on wheels). Moreover, 21% had received equipment or technology to help them maintain their independence. To maximize physical wellbeing, 20% had received advice and support to keep active, eat well, or prevent falls, 11% had received support for their physical health, and 13% of people with dementia had received help with pain management.

Regarding making decisions about future care, 43% had received information or support to put in place a lasting power of attorney, and significantly more people had received this in rural areas (*p* < 0.001). Information and support around advanced decisions to refuse treatments were given to 15%, with significantly more offered in rural areas (*p* = 0.007). Support and information around advance care planning were provided to 5% of respondents, while 13% of respondents were offered advocacy support.

#### 3.2.4. Meeting Specific Needs

Other difficulties faced by people living with dementia included sensory loss (*n* = 28), communication difficulties (*n* = 41), and impaired mobility (*n* = 40). Of those who reported additional challenges, 26 said that they were offered specific help for those challenges. This help included hearing aids, disability aids, support from charities or support groups, and support from allied health professionals. 

Contact with allied health professionals reported by respondents included occupational therapy (31%), physiotherapy (14%), and communication support (speech and language therapy; 15%). Both physiotherapy and occupational therapy were significantly higher for those in rural areas. Communication support, such as speech and language therapy was provided to 15% of respondents, and the proportion was significantly higher (*p* = 0.05) for people living with rarer forms of dementia. 

The number of people who were first-language Welsh, with Welsh as their preferred language for communication in support, was low (*n* = 12). Of these, 17% (*n* = 2) were always offered support in Welsh (in line with the Active Offer).

### 3.3. Requirements

Respondents made several recommendations for improving diagnosis and post-diagnosis support. Some suggested improvements that would require changes in how services operate, whereas others made suggestions that would be attainable without restructuring or a high financial impact.

Communication and the need for education were prominent themes. Regarding communication, people asked for sympathy, compassion, and respect at diagnosis. A need was also described in acknowledging the value of carer/family input, and there were calls for professionals to listen to the insight and knowledge of close family members, as well as include them in the process and support team from the beginning. 

People said they would like more face-to-face discussions, and tailored information that feels relevant, given at the right time. Some suggested this should take place after the ‘initial blow of the diagnosis has ebbed’. They described a need for education and advice around how to prepare and what to expect, including for example, the opportunity to ask questions, and to receive information about signposting, lasting power of attorney, plans for future care, training on how to communicate well with someone living with dementia, and how to respond to behavioral psychological symptoms of dementia. People also said that they would benefit from counseling or someone to talk to (for family, couples, and/or individuals living with the diagnosis). 

Education for professionals who work to support people living with dementia was recommended. Suggestions for staff training on dementia and how to communicate with people living with dementia and their families were put forward, particularly around empathy, sympathy, and compassion. Furthermore, people affected by rare forms of dementia have called for increased education for health professionals about rare dementias to raise awareness and understanding, which should in turn assist with recognizing and identifying dementia, as well as in providing the most suitable support. 

At a service level, people described a need for earlier, simpler, and quicker diagnosis, with support thereafter. Respondents expressed a need for more integrated services and continued information/support from a variety of sources. Many respondents suggested that people should have a named person that they can contact, who stays in touch and helps coordinate all support. The qualitative data also revealed requests for better provision of services in Welsh and more Admiral Nurses in Wales. They also called for support to help the person with dementia have a good quality of life. 

Carers have called for more day-to-day and practical home support for people with dementia, as well as enhanced respite support. Carers also described a financial need to assist in their caring role, both in terms of knowing what support is available and how to obtain it, but also that they should receive more.

## 4. Discussion

This study explored the personal experiences of people affected by dementia in Wales, in relation to the aims around diagnosis and post-diagnostic support of the Dementia Action Plan for Wales [25]. Respondents from across Wales who are affected by both typical and rarer types of dementia, and living in both rural and urban areas, completed a survey regarding their diagnosis, support provided, and their opinions of the support received. Findings suggest both positive and negative experiences, reflecting the aforementioned ‘postcode lottery’ of service provision that the All-Wales Dementia Care Pathway of Standards [28] aims to resolve.

The findings revealed that the journey to diagnosis can be complex and difficult. Accounts were given whereby people felt that they were not being listened to or were misdiagnosed and treated for the wrong illness (e.g., depression), leading to a longer wait for diagnosis. This is consistent with findings from elsewhere in the UK [16]. Moreover, only 20% of respondents (*n* = 11) said that the diagnosis had taken place within the 12-week target of the Welsh Government [25,28].

Respondents suggested a need for the provision of appropriate training within the professional dementia care workforce, supporting Yates et al. [1]. Respondents have called for improvements in recognizing and identifying dementia, as well as in valuing the concerns, knowledge, and input of carers in this process. The guidance provided by the ‘Good Work: Dementia Learning and Development Framework’ [31] is consistent with these suggestions. Moreover, other recommendations included education for staff in communication with people with dementia and their families, particularly regarding empathy and compassion at diagnosis. An example of guidance around sharing a diagnosis of dementia is available from Forward with Dementia (www.forwardwithdementia.org, accessed on 1 November 2023). After diagnosis, people report not knowing what to do or whom to turn to, and often say they feel alone and ‘left to get on with it’, with no or very little support thereafter. Only 34% of respondents said they had a support worker, and, consistent with the previous findings of the Alzheimer’s Society [26], around one quarter (26%) reported having a personal care plan. More face-to-face discussion is desired, as well as tailored information, with some suggesting that this should take place after the ‘initial blow of the diagnosis has ebbed’. This is consistent with the review of Yates et al. [1], who propose that disclosure of the diagnosis should be a process including follow-up appointments, and with Hagan [32] who suggests that “due to disorienting feelings, one diagnostic consultation is insufficient to explain both the diagnosis and offer follow-up support” (p. 1170).

The ‘postcode lottery’ identified in this study reveals an inequity in access to care and support. Despite there being few significant differences in the rural–urban and rare-typical statistical analyses, possibly due to subsamples being small, qualitative data suggest that difficulties are present. People in rural areas described difficulties because of where they lived often due to lack of services and support nearby. Previous research suggests that this can lead to a heavier reliance on family and friends (e.g., [6,17]). Although, as described above, people seek more face-to-face support, providing resources online may partially improve accessibility for people in rural areas, provided these are co-produced with the audiences they seek to serve. These may include support via videoconference, and other online support programs and packages designed by specialists located in non-local areas. Online support interventions can improve psychological well-being, with those comprising multiple components yielding the best results [33].

Despite the results demonstrating difficulties for people living in rural areas, interestingly, the analysis revealed significantly more access to allied health professionals (AHPs) and support with planning ahead (advanced decisions and lasting power of attorney) by respondents in rural areas. Although an unexpected finding, this may be due to differences in how services are organized in rural areas. For example, it may be that more ‘health hubs’ exist in rural areas, where GP surgeries have in-house access to support such as occupational therapists, physiotherapists, and others (e.g., advice and support services). This would need investigating further but shows a promising learning opportunity for service commissioners in terms of encouraging patient access to a multidisciplinary team. In the UK, both the Scottish and Welsh governments [34,35] have developed frameworks for integrating the contribution of allied health professionals to dementia care to maximize the impact of their work. The incorporation of AHPs into post-diagnostic support encourages a more holistic strength-based approach that focuses more on what people can do [35], a factor that is associated with maintaining resilience in people living with dementia [36].

A desire for education for people living with dementia and their families is described within the present study. People describe a need for help coming to terms with the diagnosis and wanting education or training in how to prepare for the future, echoing the findings of Innes et al. [12]. This type of learning may be well-received if co-produced and delivered in partnership with people who have lived experience of the process, and much can be learned from examples of good practices that are already in place. For example, in some parts of the UK, in-person ‘A Good Life with Dementia’ courses have been created and run by people living with dementia for people who have recently been diagnosed with dementia, aimed at talking to people about their diagnosis and the future [37,38]. Online and internationally available, the ‘Forward with Dementia’ website (www.forwardwithdementia.org) developed with, and for, people living with dementia and carers, offers information and toolkits for people with dementia, unpaid carers, care workers, and health care professionals.

A key aspect of any co-produced education or training package/course for people affected by dementia is highlighting the benefits of peer support. This was emphasized in the qualitative data of the present study also, with those who had experienced peer support describing the impact on their lives; a contrasting experience to those who reported feeling alone. Peer support has been associated with alleviating several of the requirements reported in the present study such as advice and information, improving understanding of dementia, sharing experiences, and learning strategies, feeling less alone, feeling listened to, and feelings of belonging and empathy [39,40,41]. Therefore, increasing awareness of, and ensuring access to, appropriate peer support opportunities should be a priority.

People reporting receipt of different types of post-diagnostic support are low (Table 5). It is not clear whether some of these supports were available but people were not referred to them, or that people were unaware they were on offer; or whether they simply were not available, wanted, or needed. However, these low figures combined with reports of respondents having to ‘fight’ and do their own research to find suitable support suggests that at least partially some support may be available, and there is a need to make accessing them as easy as possible. People need information, signposting, and support at the right time to live as well as possible throughout their journey. Many ask for a named contact, which is in line with findings from a review of the international literature that a having person who is a “point of entry” can facilitate access to services [17]. With the right education, training, and research of local support services, networks, and organizations, professional awareness could be improved. Dementia support workers, who have a greater role in post-diagnostic support may be well-placed to share this information with people affected by dementia.

There appear to be additional difficulties for people affected by rarer forms of dementia, who describe a lack of understanding and awareness leading to difficulties obtaining a diagnosis and adequate support thereafter. This supports Millenaar et al. [14] who found that for people with young onset dementia internationally, the diagnostic process and finding subsequent support was often challenging. Furthermore, a report by the Alzheimer’s Society [13] revealed a lack of awareness around younger onset dementias by healthcare professionals in Wales. Education around rare dementias was recommended by respondents, and future health and care staff educational packages may wish to utilize resources that are already in place around rare dementias, such as The Many Faces of Dementia online course (www.futurelearn.com/courses/faces-of-dementia, accessed on 1 November 2023). In addition to the difficulties around awareness and understanding from professionals, it was found that people with rare forms of dementia are also required to travel further for specialist support, and those living in rural areas described a lack of appropriate nearby services and supports, meaning they face multiple barriers to accessing suitable support.

Unpaid carers are a significant part of the dementia care workforce, and the evidence suggests that they are under-supported in their role. Carers made attainable recommendations, which would ultimately likely be cost-effective and reduce the strain on formal services. Suggestions included education and training for carers in preparing for the future, carers being treated as part of the support team, provision of practical help to support the person with dementia to remain at home as long as possible, help accessing financial support, counseling, and appropriate respite. All of these are aims within the current policy documents [25,27,28]. While most of these requirements would require funding for additional service and staff time, it would be beneficial to signpost carers to the freely available ‘iSupport’ [42], a skills and training manual for carers of people living with dementia that has been adapted for use in 41 countries.

Finally, Wales has two official languages, English and Welsh, and the ‘Active Offer’ legislation in place means that services should be offered in Welsh without people having to ask. However, people who are first-language Welsh often do not obtain access to Welsh language appointments. First-language Welsh speakers are unlikely to have the same experience in any appointment if they cannot speak the language that comes most naturally to them. While recruitment of more Welsh-speaking staff will be necessary for consistent delivery of the Active Offer, recording language preferences could help ensure suitable language provisions are made ahead of pre-booked appointments enabling the person to speak their preferred language.

### Limitations

There are limitations related to adopting a survey design. The study advert was circulated widely to reach a large and geographically distributed sample and to attempt to minimize biases in respondents. However, we acknowledge that by adopting a survey design, selection bias may exist due to the potentially non-representative nature of those with internet access, as well as those who self-select to participate in research [43]. We also recognize the limitations in the appeal of a survey that is long and only available online. Moreover, despite recruiting via networks that include people living with dementia across Wales, the number of people living with dementia who completed the survey was low (*n* = 10). Recruiting people with lived experience of dementia can be difficult, and small sample sizes are common [12,44,45]. Despite this limitation, the questions explored the experience of the person with dementia, even if filled in by the carer and, therefore, still provide some insight into the lived experience by proxy. However, we acknowledge that assessments by proxy can bias responses [46,47]. Future work must ensure that people, particularly those living with dementia, are supported to take part in whichever way they find most accessible, for example by offering paper versions and in-depth semi-structured interviewing if preferred. 

By making the survey available online and open to anyone affected by dementia in Wales, we had hoped to attract a large and diverse sample of respondents. However, there were no respondents from Black, Asian, and minority ethnic backgrounds. This lack of diversity is common in dementia research [48] and indicates a need to find ways to reach out to and include people from diverse backgrounds. Efforts should include community engagement and developing meaningful partnerships with organizations beyond academia [49].

This study importantly includes input from people affected by rare dementias. A subsample of 17 respondents provides valuable information about the experiences of people with rare forms of dementia, however, this sample size is too small to generalize the findings. Further, such a small subsample may often have been too small to make any statistical inference. Furthermore, we did not correct for multiple comparisons in the statistical analyses due to the exploratory nature of the work and acknowledge that, while aiming to avoid type 2 errors, we may have increased the type 1 error rate. 

## 5. Conclusions

The policies and legislation in place in Wales are progressive but may not yet have the desired impact. There is regional work going on to address the imbalances in access to care and support but there is still variation in how services are commissioned and how they operate, meaning that there is variation in access and quality of care and diagnosis. Integration across services and sectors will be essential for delivering support right across the pathway, from pre-diagnosis to end-of-life. The development of the next iteration of the Dementia Action Plan for Wales may wish to consider the issues highlighted by people with lived experience in this paper. However, despite there being areas needing improvement, it is important to emphasize that good work is also happening in Wales to support people living with dementia. For example, the praise for some organizations and charities, and specific individuals within them should be celebrated. These individuals have made a marked difference to the experiences of the respondents, which demonstrates the impact that an individual can have provided they are equipped with the right education and approach to supporting people living with dementia and their families. 

Many of the examples of good practice and proposed requirements highlighted by respondents in the present study are supported by a recent study reporting the key components for good post-diagnostic support of people affected by dementia in England and Wales [50]. Moreover, while the present study focused on the experiences and wishes of people in Wales, the findings around the diagnosis and post-diagnosis support reported in this paper largely reflect perceptions and needs of people living with dementia internationally [14,17,51,52,53] and, thus, provide a learning opportunity for policymakers further afield.

## Figures and Tables

**Table 1:** Demographic and diagnosis information about people living with dementia and carers.

			Person Completing Survey	
		Totals	Carer (*n* = 61)	Person with dementia (*n* = 10)
**Carer**				
Gender				
	Male	6	6	n/a
	Female	55	55	n/a
Age at the time of survey (years)				
	Mean (SD)	59.29 (12.43)	59.29 (12.43)	n/a
	Range	29–78	29–78	n/a
Relationship to PLWD				
	Their spouse/partner	26	26	n/a
	Their child	31	31	n/a
	Their parent	2	2	n/a
	Missing data	2	2	n/a
Ethnicity				
	British	58	58	n/a
	Missing data	3	3	n/a
Carer occupation				
	Retired	23	23	n/a
	Retired on medical grounds	3	3	n/a
	Long-term sick or disabled	3	3	n/a
	Employed/self-employed	23	23	n/a
	Other: full-time carer	7	7	n/a
	Student	1	1	n/a
	Missing data	1	1	n/a
**Person with dementia**				
Gender				
	Male	39	32	7
	Female	32	29	3
Age at the time of survey (years)				
	Mean	75.7 (8.6)	77.5 (7.57)	65.9 (7.98)
	Range	55–93	59–93	55–80
Marital status				
	Married/cohabiting	44	37	7
	Divorced/separated	7	4	3
	Single	1	1	0
	Widowed	18	18	0
	Missing data	1	1	0
Ethnicity				
	British	66	57	9
	Any other white background	3	2	1
	Missing data	2	2	0
PLWD Occupation				
	Retired	56	52	4
	Retired on medical grounds	7	2	5
	Long-term sick or disabled	4	3	1
	Employed/self-employed	1	1	0
	Deceased	3	3	0
Living arrangements of the person with dementia				
	Lives alone	16	11	5
	Lives with spouse/partner	36	32	4
	Lives with child(ren)	9	8	1
	Care/nursing home	9	9	0
	Missing data	1	1	0
Location of residence				
	Rural/semi-rural	37	31	6
	Urban/suburban	34	30	4
Native language				
	English	56	47	9
	Welsh	13	12	1
	Other	1	1	0
	Missing data	1	1	0
Dementia diagnoses				
	Alzheimer’s disease [AD]	22	21	1
	Vascular dementia [VD]	14	12	2
	AD and VD mixed	13	11	2
	Frontotemporal dementia [FTD]	5	3	2
	Post-cortical atrophy [PCA]	3	2	1
	Primary Progressive Aphasia [PPA]	1	0	1
	Young-onset dementia	1	0	1
	Lewy Body dementia [LBD]	2	2	0
	Logopenic progressive aphasia [LPA]	2	2	0
	Semantic dementia [SD]	1	1	0
	Familial FTD [fFTD]	1	1	0
	Parkinson’s dementia	1	1	0
	No specific diagnosis given	5	5	0

Note: n/a = not applicable.

**Table 2:** An overview of the diagnosis process experienced.

		Total *n*
How long the person with dementia has been diagnosed:		
	Less than 1 year	15
	1–2 years	11
	3–5 years	12
	6–10 years	8
	Missing data	25
When the person with dementia started noticing symptoms:		
	Less than 1 year ago	8
	1–2 years ago	12
	3–5 years ago	24
	6–10 years ago	15
	Over 10 years ago	9
	Missing data	3
Length of time from visiting GP to diagnosis:		
	0–3 months	11
	3–6 months	12
	6–12 months	15
	1–2 years	8
	More than 2 years	19
	Don’t know	2
	Missing data	4
Reason(s) for first visiting GP:		
	Worried about memory	43
	Worried about physical symptoms	12
	Referred by another professional	7
	Encouraged to go by family	32
	Appointment made by family	23
	Unrelated illness/problem	5
	Other	12
What happened on that first visit?		
	Full medical history taken	11
	Some basic physical tests	23
	Some basic memory tests	42
	Don’t know	5
	Other	13
Referral from GP to the following:		
	Memory clinic	36
	Old age/adult psychiatrist	3
	Hospital	3
	Neurologist	1
	Geriatrician	1
	Another GP appointment	3
	Brain scan (CT/MRI)	8
	Other	14
	Missing data	2
How the diagnosis was delivered:		
	Face to face	58
	Phone	5
	Email or letter	3
	Online (e.g., Zoom)	1
	Missing data	4
Was the diagnosis given verbally and in writing?		
	Yes	39
	No	21
	Don’t know	6
	Missing data	5
Who attended the diagnosis appointment with the person with dementia?		
	Person with dementia alone	10
	Person with dementia alone due to COVID restrictions	3
	Spouse	30
	Child	11
	Family member	4
	Spouse and child	1
	Missing data	12

**Table 3:** Personal perspectives around assessment and diagnosis.

		Total *n* (%)		
	*n*	Agree	Neutral	Disagree
The diagnosis was given with empathy	*67*	41 (61%)	13 (19%)	13 (19%)
Everything was explained clearly to me/us	*67*	38 (57%)	10 (15%)	19 (28%)
I/we understood the symptoms	*68*	42 (62%)	12 (18%)	14 (20%)
The person giving the diagnosis was helpful	*65*	36 (55%)	17 (26%)	12 (18%)
The person giving the diagnosis knew what support was available	*67*	23 (34%)	18 (27%)	26 (39%)
I/we knew where to go for help	*65*	19 (29%)	10 (15%)	36 (55%)
The diagnosis was given at the right time	*66*	30 (45%)	13 (20%)	23 (35%)
I/we knew what was going to happen next	*67*	18 (27%)	16 (24%)	33 (49%)

**Table 4:** Personal perspectives around post-diagnosis support.

		Total *n* (%)		
	*n*	Agree	Neutral	Disagree
I have support that helps me live my life	*71*	40 (56%)	13 (18%)	18 (25%)
I know services are designed around me and my needs	*69*	19 (28%)	20 (29%)	30 (43%)
I have personal choice and control or influence over decisions about me	*69*	35 (51%)	13 (19%)	21 (30%)
I have a sense of belonging and being valued, part of family, community, and civic life	*71*	39 (54%)	16 (23%)	16 (23%)
I live in a supportive environment where I feel valued and understood	*71*	44 (62%)	16 (23%)	11 (15%)

**Table 5:** Support received by people living with dementia in Wales.

	Total *n* (%)	*X* ^2^
A personal care plan (for a person with dementia)	17/65 (26%)	ns
A support worker (for a person with dementia)	22/65 (34%)	ns
* Do they connect you with appropriate support and information?	10/21 (48%)	ns
* If no, have you been offered a support worker?	1/36 (3%)	ns
A carer needs assessment (for carers only)	16/60 (27%)	ns
Support offered in your preferred language at all times.	45/65 (69%)	ns
Support offered in your preferred language, with Welsh being the preferred language.	2/12 (17%)	ns
Leaflets/written information to review	50/61 (82%)	ns
Contact information for relevant charities	44/61 (72%)	ns
Details of support groups e.g., dementia cafes	41/61 (67%)	ns
Support from the Dementia Helpline	6/64 (9%)	ns
Support to help you live at home, e.g., home care, meals on wheels	12/61 (20%)	ns
Advice about adjustments to your environment (e.g., home)	28/61 (46%)	ns
Help with keeping active, eating well, or preventing falls	12/61 (20%)	ns
Support for your physical health	7/61 (11%)	ns
Help with pain management	8/61 (13%)	ns
Support to help improve and maintain your memory	15/61 (25%)	ns
Support to improve/maintain quality of life (e.g., art, music, sport, reminiscence groups)	18/61 (30%)	ns
Financial support	24/61 (39%)	ns
Information and opportunity to make decisions about future care (e.g., lasting power of attorney)	26/61 (43%)	R **
Advanced decisions to refuse treatments	9/61 (15%)	R *
Advanced care planning	3/61 (5%)	ns
Help with equipment or technology that helps you keep your independence	13/61 (21%)	ns
Respite support that suits your needs	10/61 (16%)	ns
Advocacy services (someone who will speak on your behalf)	8/61 (13%)	ns
Opportunities to take part in research	15/61 (24%)	ns
Specific help for any additional needs (e.g., sensory, communication, mobility)	28/59 (47%)	ns
Occupational therapy (Allied Health)	20/64 (31%)	R *
Physiotherapy (Allied Health)	9/65 (14%)	R *
Communication support (e.g., speech and language therapy)	9/61 (15%)	RD *
Counseling support	8/61 (13%)	RD *
Do you think that your experience might be difficult because of where you live?	17/67 (25%)	RD *

Note: *p* < 0.001 marked with **, *p* < 0.05 marked with *; significantly higher rural compared to urban = R; Significantly higher rare dementia compared to typical = RD; no significant differences found = ns.

## Data Availability

Data from this study are stored in a secure data repository at UCL; the Data Safe Haven. The datasets for this study are available upon reasonable request.

## Supplementary Materials

The following supporting information can be downloaded at https://www.mdpi.com/article/10.3390/ijerph21060709/s1, Supplementary Material S1: Person living with dementia questionnaire and Carer questionnaire; Supplementary Material S2: Additional information Tables S1–S3.
