# Supplementary material for: “It’s a Postcode Lottery”: How Do People Affected by Dementia in Wales Experience Their Diagnosis and Post-Diagnostic Support, and How May These Be Improved?
Source: Int J Environ Res Public Health. 2024 May 30;21(6):709. doi: 10.3390/ijerph21060709 (PMC11203760; doi:10.3390/ijerph21060709)
Supplement: Supplementary file 1 [file ijerph-21-00709-s001.zip › Supplementary Material S1_Person_with_dementia_and_Carer_questionnaires.pdf]

Supplementary Material: Person with dementia questionnaire

**Question**	**Response options**
Full Name	
If someone is helping you to complete this questionnaire, what help are they giving?	1. Reading out questions<br>2. Support/Companionship<br>3. N/A<br>4. Other (please specify)
Please select an option below. Are you a:	**1. Person living with dementia**<br>2. Family member/carer/friend of someone living with dementia/bereaved carer
Gender	1. Male<br>2. Female<br>3. Prefer not to say
Date of Birth	DD/MM/YYYY
Are you married/single/widowed or divorced? (if no, are you separated or cohabiting)	1. Married<br>2. Cohabiting<br>3. Single<br>4. Widowed<br>5. Divorced/separated<br>6. Prefer not to say
Do you live with others?<br><br>If yes: Who do you live with?	1. Yes<br>2. No<br><br>1. Spouse/partner<br>2. Child/children<br>3. Parents<br>4. Other (please specify)
What is your postcode?	
How would you describe where you live?	1. Rural<br>2. Urban<br>3. Other (please specify)
What community resources are available where you live? Please tick all that apply:	1. Small shops<br>2. Supermarket<br>3. Post office<br>4. Community centre<br>5. Library<br>6. Public transport<br>7. GP surgery<br>8. Hospital<br>9. Pub(s)<br>10. Café(s)<br>11. Garage/petrol station<br>12. Park<br>13. School<br>14. Church/chapel<br>15. Any other resources you think are important about where you live (please specify)

Do you use these resources?	1. Yes<br>2. No
If no, did you use these resources in the past?	1. Yes<br>2. No
How would you describe your ethnic group?	1. English/Welsh/Scottish/Northern Irish/British<br>2. Any other white background<br>3. Irish<br>4. Gypsy or Irish traveller<br>5. White and black Caribbean<br>6. White and black African<br>7. White and Asian<br>8. Any other mixed/multiple ethnic backgrounds<br>9. Indian<br>10. Pakistani<br>11. Bangladeshi<br>12. Chinese<br>13. Any other Asian background<br>14. African<br>15. Caribbean<br>16. Any other Black/African/Caribbean background<br>17. Arab<br>18. Prefer not to say<br>19. Any other ethnic group (please specify)
What is your native language?	1. English<br>2. Welsh<br>3. Other language (but having good knowledge of English/Welsh)<br>4. Other language (and having poor or no knowledge of English/Welsh)
What option best describes your current occupation?	1. Employed or self employed<br>2. Retired<br>3. Retired on medical grounds<br>4. Seeking work<br>5. Looking after home/family<br>6. Long term sick or disabled<br>7. Student (full time)<br>8. Other e.g. full time carer (specify)
What was/is your specific job/title?	

**Your diagnosis and the support that you have received.**	
**Please tell us how much you agree or disagree with these statements:**	
I have support that helps me live my life	1. Strongly Agree<br>2. Agree<br>3. Neither Agree nor Disagree<br>4. Disagree<br>5. Strongly Disagree<br>6. Don't Know

Your diagnosis and the support that you have received.	
I know services are designed around me and my needs	1. Strongly Agree<br>2. Agree<br>3. Neither Agree nor Disagree<br>4. Disagree<br>5. Strongly Disagree<br>6. Don't Know
I have personal choice and control or influence over decisions about me	1. Strongly Agree<br>2. Agree<br>3. Neither Agree nor Disagree<br>4. Disagree<br>5. Strongly Disagree<br>6. Don't Know
I have a sense of belonging and being valued, part of family, community and civic life	1. Strongly Agree<br>2. Agree<br>3. Neither Agree nor Disagree<br>4. Disagree<br>5. Strongly Disagree<br>6. Don't Know
I live in a supportive environment where I feel valued and understood	1. Strongly Agree<br>2. Agree<br>3. Neither Agree nor Disagree<br>4. Disagree<br>5. Strongly Disagree<br>6. Don't Know
**The next questions are about your diagnosis:**	
Were you diagnosed with a specific type of dementia?<br><br>If yes:<br>What type of dementia has been diagnosed?	1. Yes<br>2. No<br><br><br>1. Alzheimer's Disease (AD)<br>2. Vascular dementia (VD)<br>3. lewy body dementia (LBD)<br>4. familial Alzheimer's disease (fAD)<br>5. familial frontotemporal dementia (fFTD)<br>6. frontotemporal dementia (FTD)<br>7. primary progressive aphasia (PPA)<br>8. logopenic aphasia (LPA)<br>9. progressive nonfluent aphasia (PNFA)<br>10. semantic dementia (SD)<br>11. posterior cortical atrophy (PCA)<br>12. AD&VD<br>13. fFTD&PPA<br>14. Other (please specify)
How long have you been diagnosed with dementia?	1. Less than 6 months<br>2. 6-12 months<br>3. 1-2 years<br>4. 2-3 years

Your diagnosis and the support that you have received.	
	5. 3-4 years<br>6. 4-5 years<br>7. 5-6 years<br>8. 6-7 years<br>9. 7-8 years<br>10. 8-9 years<br>11. 9-10 years<br>12. More than 10 years
When did you first start noticing symptoms?	1. Less than one year ago<br>2. 1-2 years ago<br>3. 3-5 years ago<br>4. 6-10 years ago<br>5. Over 10 years ago
Thinking about your dementia diagnosis, why did you first visit your GP? (Tick all that apply)	1. I was worried about my memory<br>2. I was worried about some physical symptoms<br>3. I was referred by another professional (e.g. Nurse, Social Worker)<br>4. I was encouraged to go by a family member<br>5. Appointment was made by a family member<br>6. Another unrelated illness/problem<br>7. I don't know<br>8. Other (please specify)
What happened on that first visit? (Tick all that apply)	1. Full medical history taken<br>2. Some basic physical tests were performed (e.g. blood test)<br>3. Some basic memory tests were performed<br>4. Don't know<br>5. Other (please specify)
How many times did you speak to your GP before you were referred to a memory clinic?	Tab displaying 1-10+<br>Other (please specify)
Roughly how many times have you spoken to your GP in the last year?	Tab displaying 1-10+<br>Other (please specify)
Do you get to speak to the same GP every time?	1. Yes always<br>2. Most of the time<br>3. Sometimes<br>4. Not often<br>5. Never
What happened after you first visited your GP? Were you referred elsewhere? Where were you referred?	1. Referred to a memory clinic/service<br>2. Referred to a psychiatrist (general)<br>3. Referred to an old age psychiatrist<br>4. Referred to an adult psychiatrist<br>5. Referred to the hospital<br>6. Referred to a neurological clinic/neurologist<br>7. Referred to a geriatrician

Your diagnosis and the support that you have received.	
	8. Told to make another appointment with the GP<br>9. Referred for a brain scan (CT/CAT or MRI)<br>10. Don't know<br>11. Other (please specify)
If yes, roughly how many times did you see that person?	Tab displaying 1-10+<br>Other (please specify)
Who was the first to think this might be a dementia?	1. GP<br>2. Neurologist<br>3. Psychiatrist<br>4. Other healthcare professional e.g. optometrist<br>5. Family member<br>6. Friend<br>7. Yourself (person with dementia)<br>8. Carer<br>9. Other (please specify)
Approximately how long did you have to wait between your first visit to the GP and receiving your dementia diagnosis? (Tick one box)	1. Less than a month<br>2. Between 1 and 2 months<br>3. Between 2 and 3 months<br>4. Between 3 and 6 months<br>5. Between 6 and 12 months<br>6. Between 1 and 2 years<br>7. More than 2 years<br>8. Don't know
Where were you diagnosed?	1. GP surgery<br>2. memory clinic<br>3. community mental health team<br>4. during hospital stay<br>5. Other (please specify)
Did someone come with you?	1. Yes<br>2. No<br>3. Covid restrictions did not allow anyone to come with me
*if yes, who?	1. Spouse<br>2. Child<br>3. Family member<br>4. Friend<br>5. Other – please specify
How was your diagnosis given to you?	1. Face to face<br>2. Over the phone<br>3. Via email/letter<br>4. Online (e.g. via Zoom)

Your diagnosis and the support that you have received.	
Did you receive your diagnosis both verbally and in writing? (Tick one box)	1. Yes<br>2. No<br>3. Don't know
How far do you agree or disagree with the following statements about what happened at or around the time of diagnosis...?<br>**Statements:**<br>• The diagnosis was given with empathy<br>• Things were explained to me<br>• I understood the symptoms<br>• The person giving the diagnosis was helpful<br>• The person giving the diagnosis knew what support was available for me<br>• I knew where to go for help<br>• The diagnosis was given at the right time<br>• I knew what was going to happen next	1. Strongly agree<br>2. Agree<br>3. Neither agree nor disagree<br>4. Disagree<br>5. Strongly disagree<br>6. N/A
How would you rate the overall process of diagnosis from when you first saw your GP to when you received your final diagnosis?	1. Very good<br>2. Good<br>3. Neither good nor poor<br>4. Poor<br>5. Very poor<br>6. Don't know
How far do you have to travel for the following:<br><br>GP visits<br>Hospital visits<br>Memory clinic<br>Specialist centres	1. less than 10 miles<br>2. 10-20 miles<br>3. 20-40 miles<br>4. 40-60 miles<br>5. 60-100 miles<br>6. 100-200 miles<br>7. more than 200 miles
Do you think that your experience might be difficult/different because of where you live? If yes, why so?	1. Yes (please specify)<br>2. No
Which language do you prefer to speak in appointments and healthcare?	1. English<br>2. Welsh<br>3. Other (please specify)
Are you offered support in your preferred language?	1. Yes<br>2. Sometimes<br>3. No

Your diagnosis and the support that you have received.	
Do any of the following apply to you?<br>• sensory loss (e.g. hearing or vision loss)<br>• communication difficulties<br>• mobility difficulties<br>• learning disabilities<br><br>If yes, have you been offered any specific help for this?	1. yes<br>2. no<br><br><br><br><br>1. Yes (please specify)<br>2. No
Which of the following have you received since the diagnosis? (Tick all that apply)	1. Leaflets/written Information for you and your family to review<br>2. Contact information for relevant charities<br>3. Details of support groups e.g. dementia cafes<br>4. Support to help you live at home e.g home care, meals on wheels<br>5. advice about adjustments to your environment (e.g. home)<br>6. Support to help improve and maintain your memory<br>7. Support to improve or maintain quality of life e.g. art, music, sport, reminiscence groups<br>8. Financial support<br>9. Support for your physical health<br>10. Help with keeping active, eating well or preventing falls<br>11. Help with pain management<br>12. Communication support, such as speech and language therapy.<br>13. Counselling support.<br>14. Information and opportunity to make decisions about your own future care (e.g. lasting power of attorney)<br>15. Advanced decisions to refuse treatments<br>16. Advanced care planning<br>17. Help with equipment or technology that help you keep your independence<br>18. Respite support that suits your needs<br>19. Advocacy services (someone that will speak on your behalf)<br>20. Opportunities to take part in research
Do you have a Personal Care Plan?	1. Yes<br>2. No<br>3. Don't know

Your diagnosis and the support that you have received.	
***if yes:** How far do you agree or disagree with the following statements about your Personal Care Plan?<br>**Statements:**<br>• I felt involved in drawing up my care plan<br>• My carer was involved in drawing up my care plan<br>• My care plan reflects my goals<br>• My care plan is clear and easy to understand<br>• My care plan is helpful	1. Strongly agree<br>2. Agree<br>3. Neither agree nor disagree<br>4. Disagree<br>5. Strongly disagree<br>6. N/A
Do you have a support worker (one named person that you can contact about your care or anything you're worried about)?	1. Yes<br>2. No<br>3. Don't know
*If yes, do they connect you with support and information that is appropriate to you?	1. Yes<br>2. A little bit<br>3. No
*if yes, how often do you speak to your support worker?	1. Every week<br>2. Every couple of weeks<br>3. Every month<br>4. Every couple of months<br>5. Every 6 months<br>6. Other (please specify)
*If no, have you been offered a support worker?	1. Yes<br>2. No<br>3. Don't know
Since the diagnosis how would you rate the support you've received from the following people?<br>**Statements:**<br>• NHS staff<br>• Staff at your GP surgery<br>• Staff within your local hospital<br>• People within local support charities<br>• People within national charities<br>• Staff from social services<br>• Support worker<br>**local charities= services specifically in your area e.g. local AgeUK*<br>**national charities= e.g. dementia helpline*	1. Very good<br>2. Good<br>3. Neither good nor poor<br>4. Poor<br>5. Very poor<br>6. N/A
Overall, how would you rate the support you've received since your diagnosis?	1. Very good<br>2. Good<br>3. Neither good nor poor<br>4. Poor<br>5. Very poor<br>6. Don't know
Which of these health professionals have you had contact with (please tick all that apply):	1. Psychiatrist

Your diagnosis and the support that you have received.	
	2. Geriatrician<br>3. Neurologist<br>4. Consultant at the memory clinic<br>5. Mental health nurse<br>6. Community psychiatric nurse (CPN)<br>7. Psychologist<br>8. Occupational therapist<br>9. Social worker<br>10. Physiotherapist<br>11. Dementia support worker<br>12. Dementia advisers (professionals who provide information, advice and guidance to people with dementia and their carers)<br>13. Other (please specify)
*if yes, roughly how many times have you seen them?	Free text
Which organisations have you been in touch with:	1. Alzheimer's Society<br>2. Dementia helpline<br>3. Carers Trust<br>4. Age Cymru<br>5. Join Dementia Research (JDR)<br>6. Rare Dementia Support (RDS)<br>7. Admiral nurses (for carers of people living with dementia)

Helping others in the future<br>**We would like to ask your advice on what could be improved about the diagnosis experience in Wales.**<br>*Please write your responses in the space provided.*	
What has been most helpful?	Text
What has been most difficult?	Text
What could be done differently in future?	Text
What do you think would have been the most useful support to be offered immediately after your diagnosis?	Text
Is there anything about your experience you would like to see improved?	Text
If you were given the diagnosis again, is there anything about the way it was delivered that you'd suggest they change?	Text
Is there anything else that is important to you, that we have not covered?	Text

Supplementary Material: Carer's questionnaire

**Question**	**Response options**
Full Name	
Please select an option below. Are you a:	1. Person living with dementia<br>**2. Family member/carer/friend of someone living with dementia/bereaved carer**
Carer: Gender	1. Male<br>2. Female<br>3. Prefer not to say
Carer: Date of Birth	DD/MM/YYYY
Carer: Ethnicity	1. English/Welsh/Scottish/Northern Irish/British<br>1. Irish<br>2. Gypsy or Irish traveller<br>3. Any other white background<br>4. White and black Caribbean<br>5. White and black African<br>6. White and Asian<br>7. Any other mixed/multiple ethnic backgrounds<br>8. Indian<br>9. Pakistani<br>10. Bangladeshi<br>11. Chinese<br>12. Any other Asian background<br>13. African<br>14. Caribbean<br>15. Any other Black/African/Caribbean background<br>16. Arab<br>17. Prefer not to say<br>18. Any other ethnic group (specify)
Carer: Marital status	1. Married<br>2. Cohabiting<br>3. Single<br>4. Widowed<br>5. Divorced/separated<br>6. Prefer not to say
Carer: Occupation	1. Employed or self employed<br>2. Retired<br>3. Retired on medical grounds<br>4. Seeking work<br>5. Looking after home/family<br>6. Long term sick or disabled<br>7. Student (full time)<br>8. Other e.g. full time carer (specify)
Carer: Relationship to the person with dementia	1. Spouse/partner<br>2. Their child<br>3. Their parent
Carer: Have you received an assessment of your needs as a carer?	1. Yes<br>2. No<br>3. don't know

**Information about the person with Dementia:**	
Gender	1. Male<br>2. Female<br>3. Prefer not to say
Date of Birth	DD/MM/YYYY
Are they married/single/widowed or divorced? (if no, are you separated or cohabiting)	1. Married<br>2. Cohabiting<br>3. Single<br>4. Widowed<br>5. Divorced/separated<br>6. Prefer not to say
Do they live with you?<br><br>If no: Who do they live with?	1. Yes<br>2. No<br><br>1. Alone<br>2. Spouse/partner<br>3. Child/children<br>4. Parents<br>5. Other (please specify)
What is their postcode?	
How would you describe they live?	1. Rural<br>2. Urban<br>3. Other (please specify)
What community resources are available where they live? Please tick all that apply:	1. Small shops<br>2. Supermarket<br>3. Post office<br>4. Community centre<br>5. Library<br>6. Public transport<br>7. GP surgery<br>8. Hospital<br>9. Pub(s)<br>10. Café(s)<br>11. Garage/petrol station<br>12. Park<br>13. School<br>14. Church/chapel<br>15. Any other resources you think are important about where you live (please specify)
Do they use these resources?	1. Yes<br>2. No
If no, did they use these resources in the past?	1. Yes<br>2. No
How would you describe their ethnic group?	1. English/Welsh/Scottish/Northern Irish/British<br>2. Any other white background<br>3. Irish<br>4. Gypsy or Irish traveller

	5. White and black Caribbean<br>6. White and black African<br>7. White and Asian<br>8. Any other mixed/multiple ethnic backgrounds<br>9. Indian<br>10. Pakistani<br>11. Bangladeshi<br>12. Chinese<br>13. Any other Asian background<br>14. African<br>15. Caribbean<br>16. Any other Black/African/Caribbean background<br>17. Arab<br>18. Prefer not to say<br>19. Any other ethnic group (please specify)
What is their native language?	1. English<br>2. Welsh<br>3. Other language (but having good knowledge of English/Welsh)<br>4. Other language (and having poor or no knowledge of English/Welsh)
What option best describes their current occupation?	1. Employed or self employed<br>2. Retired<br>3. Retired on medical grounds<br>4. Seeking work<br>5. Looking after home/family<br>6. Long term sick or disabled<br>7. Student (full time)<br>8. Other e.g. full time carer (please specify)
What was/is their specific job/title?	
**The diagnosis and the support received by the person living with dementia.**	
**Please tell us how much you agree or disagree with these statements about the person living with dementia that you care for:**	
They have support that helps them to live their life	1. Strongly Agree<br>2. Agree<br>3. Neither Agree nor Disagree<br>4. Disagree<br>5. Strongly Disagree<br>6. Don't Know
They know services are designed around them and their needs	1. Strongly Agree<br>2. Agree<br>3. Neither Agree nor Disagree<br>4. Disagree<br>5. Strongly Disagree<br>6. Don't Know
They have personal choice and control or influence over decisions about them	1. Strongly Agree<br>2. Agree

	3. Neither Agree nor Disagree<br>4. Disagree<br>5. Strongly Disagree<br>6. Don't Know
They have a sense of belonging and being valued, part of family, community and civic life	1. Strongly Agree<br>2. Agree<br>3. Neither Agree nor Disagree<br>4. Disagree<br>5. Strongly Disagree<br>6. Don't Know
They live in a supportive environment where they feel valued and understood	1. Strongly Agree<br>2. Agree<br>3. Neither Agree nor Disagree<br>4. Disagree<br>5. Strongly Disagree<br>6. Don't Know
**The next questions are about the diagnosis:**	
Were they diagnosed with a specific type of dementia?<br><br>If yes:<br>What type of dementia has been diagnosed?	1. Yes<br>2. No<br><br><br>1. Alzheimer's Disease (AD)<br>2. Vascular dementia (VD)<br>3. lewy body dementia (LBD)<br>4. familial Alzheimer's disease (fAD)<br>5. familial frontotemporal dementia (fFTD)<br>6. frontotemporal dementia (FTD)<br>7. primary progressive aphasia (PPA)<br>8. logopenic aphasia (LPA)<br>9. progressive nonfluent aphasia (PNFA)<br>10. semantic dementia (SD)<br>11. posterior cortical atrophy (PCA)<br>12. AD&VD<br>13. fFTD&PPA<br>14. Other (please specify)
How long have they been diagnosed with dementia?	1. Less than 6 months<br>2. 6-12 months<br>3. 1-2 years<br>4. 2-3 years<br>5. 3-4 years<br>6. 4-5 years<br>7. 5-6 years<br>8. 6-7 years<br>9. 7-8 years<br>10. 8-9 years<br>11. 9-10 years<br>12. More than 10 years
When did they first start noticing symptoms?	1. Less than one year ago<br>2. 1-2 years ago<br>3. 3-5 years ago

	4. 6-10 years ago<br>5. Over 10 years ago
When did you first start noticing symptoms? (carer)	1. Less than one year ago<br>2. 1-2 years ago<br>3. 3-5 years ago<br>4. 6-10 years ago<br>5. Over 10 years ago
Thinking about the dementia diagnosis, why was the GP first contacted? (tick all that apply)	1. Worried about memory<br>2. Worried about some physical symptoms<br>3. Referred by another professional (e.g. Nurse, Social Worker)<br>4. Encouraged to go by a family member<br>5. Appointment made by a family member<br>6. Another unrelated illness/problem<br>7. I don't know<br>8. Other (please specify)
What happened on that first visit?	Tick all that apply:<br>1. Full medical history taken<br>2. Some basic physical tests were performed (e.g. blood test)<br>3. Some basic memory tests were performed<br>4. Don't know<br>5. Other (please specify)
How many times did you/they speak to the GP before being referred to a memory clinic?	Tab displaying 1-10+<br>Other (please specify)
Roughly how many times have they spoken to the GP in the last year?	Tab displaying 1-10+<br>Other (please specify)
Do they get to speak to the same GP every time?	1. Yes, always<br>2. Most of the time<br>3. Sometimes<br>4. Not often<br>5. Never
What happened after the first visit to the GP? Were they referred elsewhere? Where were they referred?	1. Referred to a memory clinic/service<br>2. Referred to a psychiatrist (general)<br>3. Referred to an old age psychiatrist<br>4. Referred to an adult psychiatrist<br>5. Referred to the hospital<br>6. Referred to a neurological clinic/neurologist<br>7. Referred to a geriatrician<br>8. Told to make another appointment with GP<br>9. Referred for a brain scan (CT/CAT or MRI)<br>10. Don't know<br>11. Other (please specify)
If yes, roughly how many times did they see that person?	Tab displaying 1-10+<br>Other (please specify)
Who was the first to think this might be a dementia?	1. GP<br>2. Neurologist<br>3. Psychiatrist

	4. Other healthcare professional e.g. optometrist<br>5. Family member<br>6. Friend<br>7. Person with dementia<br>8. Yourself (carer)<br>9. Other (please specify)
Approximately how long did they have to wait between the first visit to the GP and receiving the dementia diagnosis?	1. Less than a month<br>2. Between 1 and 2 months<br>3. Between 2 and 3 months<br>4. Between 3 and 6 months<br>5. Between 6 and 12 months<br>6. Between 1 and 2 years<br>7. More than 2 years<br>8. Don't know
Where were they diagnosed?	1. GP surgery<br>2. Memory clinic<br>3. Community mental health team<br>4. During hospital stay<br>5. Other (please specify)
Did someone come with them?	1. Yes<br>2. No<br>3. Covid restrictions did not allow anyone to accompany them
*if yes, who?	1. Spouse<br>2. Child<br>3. Family member<br>4. Friend<br>5. Other (please specify)
How was the diagnosis given?	1. Face to face<br>2. Over the phone<br>3. Via email/letter<br>4. Online (e.g. via Zoom)
Did they receive the diagnosis both verbally and in writing?	1. Yes<br>2. No<br>3. Don't know
How far do you agree or disagree with the following statements about what happened at or around the time of diagnosis...?<br><br>**Statements:**<br>• The diagnosis was given with empathy<br>• Things were explained to us<br>• We understood the symptoms	1. Strongly agree<br>2. Agree<br>3. Neither agree nor disagree<br>4. Disagree<br>5. Strongly disagree<br>6. N/A

• The person giving the diagnosis was helpful<br>• The person giving the diagnosis knew what support was available for us<br>• We knew where to go for help<br>• The diagnosis was given at the right time<br>• We knew what was going to happen next	
How would you rate the overall process of diagnosis from first seeing the GP to receiving the final diagnosis?	1. Very good<br>2. Good<br>3. Neither good nor poor<br>4. Poor<br>5. Very poor<br>6. Don't know
How far does the person living with dementia have to travel for the following:<br><br>• GP visits<br>• Hospital visits<br>• Memory clinic<br>• Specialist centres	1. less than 10 miles<br>2. 10-20 miles<br>3. 20-40 miles<br>4. 40-60 miles<br>5. 60-100 miles<br>6. 100-200 miles<br>7. more than 200 miles
Do you think that your experience might be difficult/different because of where you/they live? If yes, why so?	1. Yes (please specify)<br>2. No
Which language do they prefer to speak in appointments and healthcare?	1. English<br>2. Welsh<br>3. Other (please specify)
Are they offered support in their preferred language?	1. Yes<br>2. Sometimes<br>3. No
Do any of the following apply to the person you care for:<br>• sensory loss (e.g. hearing or vision loss)<br>• communication difficulties<br>• mobility difficulties<br>• learning disabilities<br><br>If yes, have they been offered any specific help for this?	1. Yes<br>2. No<br><br>1. Yes (please specify)<br>2. No
Which of the following have you/they received since the diagnosis? (Tick all that apply)	1. Leaflets/written Information to review<br>2. Contact information for relevant charities<br>3. Details of support groups e.g. dementia cafes<br>4. Support to help them live at home e.g home care, meals on wheels<br>5. Advice about adjustments to their environment (e.g. home)<br>6. Support to help improve and maintain memory

	7. Support to improve or maintain quality of life e.g. art, music, sport, reminiscence groups<br>8. Financial support<br>9. Support for your physical health<br>10. Help with keeping active, eating well or preventing falls<br>11. Help with pain management<br>12. Communication support, such as speech and language therapy.<br>13. Counselling support.<br>14. Information and opportunity to make decisions about future care (e.g. lasting power of attorney)<br>15. Advanced decisions to refuse treatments<br>16. Advanced care planning<br>17. Help with equipment or technology that help keep independence<br>18. Respite support that suits your needs<br>19. Advocacy services (someone that will speak on their behalf)<br>20. Opportunities to take part in research
Does the person with dementia have a Personal Care Plan?	1. Yes<br>2. No<br>3. Don't know
*if yes: How far would you agree or disagree with the following statements about the Personal Care Plan from the perspective of the person with dementia?<br><br>**Statements:**<br>• They felt involved in drawing up the care plan<br>• Their carer was involved in drawing up the care plan<br>• The care plan reflects their goals<br>• The care plan is clear and easy to understand<br>• The care plan is helpful	1. Strongly agree<br>2. Agree<br>3. Neither agree nor disagree<br>4. Disagree<br>5. Strongly disagree<br>6. N/A
Do they have a support worker (one named person that they/you can contact about their care or anything you're worried about)?	1. Yes<br>2. No<br>3. Don't know
*If yes, do they connect them/you with support and information that is appropriate?	1. Yes<br>2. A little bit<br>3. No
*if yes, how often do they speak to the support worker?	1. Every week<br>2. Every couple of weeks

	3. Every month<br>4. Every couple of months<br>5. Every 6 months<br>6. Other (please specify)
*If no, have they been offered a support worker?	1. Yes<br>2. No<br>3. Don't know
Since the diagnosis how would you rate the support received from the following people?<br>● NHS staff<br>● Staff at your GP surgery<br>● Staff within your local hospital<br>● People within local support charities<br>● People within national charities<br>● Staff from social services<br>● Support worker<br>**local charities= services specifically in your area e.g. local AgeUK*<br>**national charities= e.g. dementia helpline*	1. Very good<br>2. Good<br>3. Neither good nor poor<br>4. Poor<br>5. Very poor<br>6. N/A
Overall, how would you rate the support they have received since the diagnosis?	1. Very good<br>2. Good<br>3. Neither good nor poor<br>4. Poor<br>5. Very poor<br>6. Don't know
Which of these health professionals have they/you had contact with (tick all that apply):	1. Psychiatrist<br>2. Geriatrician<br>3. Neurologist<br>4. Consultant at the memory clinic<br>5. Mental health nurse<br>6. Community psychiatric nurse (CPN)<br>7. Psychologist<br>8. Occupational therapist<br>9. Social worker<br>10. Physiotherapist<br>11. Dementia support worker<br>12. Dementia advisers (professionals who provide information, advice and guidance to people with dementia and their carers)<br>13. Other (please specify)
*if yes, roughly how many times have you seen them?	
Which organisations have they/you been in touch with:	1. Alzheimer's Society<br>2. Dementia helpline<br>3. Carers Trust<br>4. Age Cymru<br>5. Join Dementia Research (JDR)

	6. Rare Dementia Support (RDS)<br>7. Admiral nurses (for carers of people living with dementia)

**Helping others in the future**	
**We would like to ask your advice on what could be improved about the diagnosis experience in Wales.**<br>*Please write your responses in the space provided.*	
What has been most helpful?	Text
What has been most difficult?	Text
What could be done differently in future?	Text
What do you think would have been the most useful support to be offered immediately after your diagnosis?	Text
Is there anything about your experience you would like to see improved?	Text
If you were given the diagnosis again, is there anything about the way it was delivered that you'd suggest they change?	Text
Is there anything else that is important to you, that we have not covered?	Text
