# Supplementary material for: “It’s a Postcode Lottery”: How Do People Affected by Dementia in Wales Experience Their Diagnosis and Post-Diagnostic Support, and How May These Be Improved?
Source: Int J Environ Res Public Health. 2024 May 30;21(6):709. doi: 10.3390/ijerph21060709 (PMC11203760; doi:10.3390/ijerph21060709)
Supplement: Supplementary file 1 [file ijerph-21-00709-s001.zip › Supplementary Material S2 Additional information Tables S1_S2_S3.pdf]

Table S1. Additional information including breakdown by location of residence and dementia type.

		Location of residence							Dementia Type						
		**Rural**			**Urban**				**Rare**			**Typical**			
	N	Agree	Neutral	Disagree	Agree	Neutral	Disagree	X^2	Agree	Neutral	Disagree	Agree	Neutral	Disagree	X^2
The diagnosis was given with empathy	67	19	8	9	22	5	4	2.48	11	3	3	30	10	10	0.12
Everything was explained clearly to me/us	67	20	4	11	18	6	8	0.85	9	1	7	29	9	12	2.63
I/we understood the symptoms	68	22	6	8	20	6	6	0.15	7	4	6	35	8	8	4.38
The person giving the diagnosis was helpful	65	18	8	8	18	9	4	1.26	11	2	4	25	15	8	2.5
The person giving the diagnosis knew what support was available	67	11	10	14	12	8	12	0.27	4	4	9	19	14	17	2.04
I/we knew where to go for help	65	12	3	19	7	7	17	2.9	2	3	11	17	7	25	2.87
The diagnosis was given at the right time	66	14	9	12	16	4	11	1.86	6	2	8	24	11	15	2.24
I/we knew what was going to happen next	67	8	8	19	10	8	14	0.85	4	3	10	14	3	23	0.89

Note: All $p > 0.05$

Table S2. Additional information including breakdown by location of residence and dementia type.

		Location of residence							Dementia Type						
		Rural			Urban				Rare			Typical			
	N	Agree	Neutral	Disagree	Agree	Neutral	Disagree	X^2	Agree	Neutral	Disagree	Agree	Neutral	Disagree	X^2
I have support that helps me live my life	*71*	21	7	9	19	6	9	0.05	12	1	4	28	12	14	2.72
I know services are designed around me and my needs	*69*	10	11	14	9	9	16	0.37	3	7	7	16	13	23	1.99
I have personal choice and control or influence over decisions about me	*69*	18	6	12	17	7	9	0.40	10	2	5	25	11	16	0.90
I have a sense of belonging and being valued, part of family, community & civic life	*71*	19	9	9	20	7	7	0.40	8	6	3	31	10	13	2.10
I live in a supportive environment where I feel valued and understood	*71*	23	7	7	21	9	4	1.03	10	4	3	34	12	8	0.11

Note: All $p > 0.05$

Table S3. Additional information including breakdown by location of residence and dementia type.

		Location of residence			Dementia type		
	N	**Rural (n)**	**Urban (n)**	X^2	**Rare (n)**	**Typical (n)**	X^2
A Personal Care Plan for person with dementia	*65*	11 (34)	6 (31)	1.42	4 (16)	13 (49)	0.02
A support worker for person with dementia	*65*	12 (34)	10 (31)	0.07	7 (16)	15 (49)	0.93
**Do they connect you with appropriate support and information?*	*21*	5 (11)	5 (10)	0.04	3 (7)	7 (14)	0.96
**If no, have you been offered a support worker?*	*36*	1 (19)	0 (17)	0.92	0 (7)	1 (29)	0.25
A carers needs assessment (for carers only)	*60*	7 (31)	9 (29)	0.55	5 (12)	11 (48)	1.73
Support in your preferred language, always?	*65*	23 (34)	22 (31)	0.08	14 (16)	31 (49)	3.33
Support in preferred language, always - Welsh preferred language?	*12*	2 (8)	0 (4)	1.20	0	2 (12)	n/a
Leaflets/written information to review	*61*	30 (35)	20 (26)	0.78	14 (16)	36 (45)	0.45
Contact information for relevant charities	*61*	25 (35)	19 (26)	0.02	11 (16)	33 (45)	0.12
Details of support groups e.g. dementia cafes	*61*	23 (35)	18 (26)	0.08	11 (16)	30 (45)	0.02
Support from the Dementia Helpline	*64*	4 (34)	2 (30)	0.49	3 (16)	3 (49)	2.6
Support to help you live at home e.g. home care, meals on wheels	*61*	5 (35)	7 (26)	1.51	5 (16)	7 (45)	1.84
Advice about adjustments to your environment	*61*	17 (35)	11 (26)	0.24	8 (16)	20 (45)	0.15
Help with keeping active, eating well or preventing falls	*61*	8 (35)	4 (26)	0.53	4 (16)	8 (45)	0.39
Support for your physical health	*61*	4 (35)	3 (26)	0.00	3 (16)	4 (45)	1.13
Help with pain management	*61*	5 (35)	3 (26)	0.10	3 (16)	5 (45)	0.6
Support to help improve and maintain your memory	*61*	11 (35)	4 (26)	2.07	5 (16)	10 (45)	0.52
Support to improve or maintain quality of life (e.g. art, music, sport, reminiscence groups)	*61*	11 (35)	7 (26)	0.15	5 (16)	13 (45)	0.03
Financial support	*61*	16 (35)	8 (26)	1.40	6 (16)	18 (45)	0.31
Information and opportunity to make decisions about future care (e.g. lasting power of attorney)	*61*	22 (35)	4 (26)	**13.75****	8 (16)	18 (45)	0.48
Advanced decisions to refuse treatments	*61*	9 (35)	0 (26)	**7.84***	4 (16)	5 (45)	1.81
Advanced care planning	*61*	3 (35)	0 (26)	2.34	2 (16)	1 (45)	2.67
Help with equipment or technology that help you keep your independence	*61*	8 (35)	5 (26)	0.12	5 (16)	8 (45)	1.28
Respite support that suits your needs	*61*	7 (35)	5 (26)	0.78	4 (16)	6 (45)	1.17
Advocacy services (someone that will speak on your behalf)	*61*	7 (35)	1 (26)	3.42	3 (16)	5 (45)	0.6
Opportunities to take part in research	*61*	11 (35)	4 (26)	2.07	6 (16)	9 (45)	1.95
Specific help for any additional needs (e.g. sensory, communication, mobility)?	*59*	16 (30)	12 (29)	0.85	11 (17)	17 (42)	2.85
Occupational Therapy (Allied Health)	*64*	15 (34)	5 (35)	**5.59***	7 (15)	13 (49)	2.17
Physiotherapy (Allied Health)	*65*	8 (34)	1 (31)	**5.6***	3 (15)	6 (50)	0.62
Communication support (e.g. speech and language therapy: Allied Health)	*61*	8 (35)	1 (26)	4.29	5 (16)	4 (45)	**4.69***
Counselling support	*61*	6 (35)	2 (26)	1.17	5 (16)	3 (45)	**6.26***
Do you think that your experience might be difficult/different because of where you live?	*67*	12 (36)	5 (31)	2.60	9 (17)	8 (50)	**9.14***

Note: p<0.001 marked with **; p<0.05 marked with *
